# Need for standardization of Influenza A virus-induced cell death in vivo to improve consistency of inter-laboratory research findings

**DOI:** 10.1038/s41420-024-01981-w

**Published:** 2024-05-22

**Authors:** Teodora Oltean, Jonathan Maelfait, Xavier Saelens, Peter Vandenabeele

**Affiliations:** 1 VIB Center for Inflammation Research, Cell Death and Inflammation Unit, Ghent, Belgium; 2 Department of Biomedical Molecular Biology, Ghent University, Ghent, Belgium; 3 VIB Center for Medical Biotechnology, Ghent, Belgium; 4 Department of Biochemistry and Microbiology, Ghent University, Ghent, Belgium

**Keywords:** Necroptosis, Microbiology, Apoptosis

## Abstract

The involvement of necroptosis in the control of influenza A virus (IAV) infection has been reported in multiple studies. Downstream of the nucleic acid sensor ZBP1, RIPK3 kinase activity is critically involved in the induction of necroptotic cell death by phosphorylating MLKL, while RIPK3 as a scaffold can induce apoptosis. Paradoxically, *RIPK3*-deficiency of mice may result in increased or decreased susceptibility to IAV infection. Here, we critically review the published reports on the involvement of RIPK3 in IAV infection susceptibility and try to identify differences in experimental settings that could explain seemingly conflicting outcomes. Analysis of the experimental reports revealed differences in the IAV challenge dose, the IAV inoculum preparation, IAV titer assessment, as well as the route of inoculation between studies. Furthermore, differences were noticed in the inclusion of littermate controls, which show high variance in viral sensitivity. Our evaluation argues for a standardized setup for IAV infection experiments including the preparation of the IAV virus, the use of different IAV infectious doses description and the proper experimental genetic controls of the mouse strains to increase inter-laboratory consistency in this field.

**Figure:**
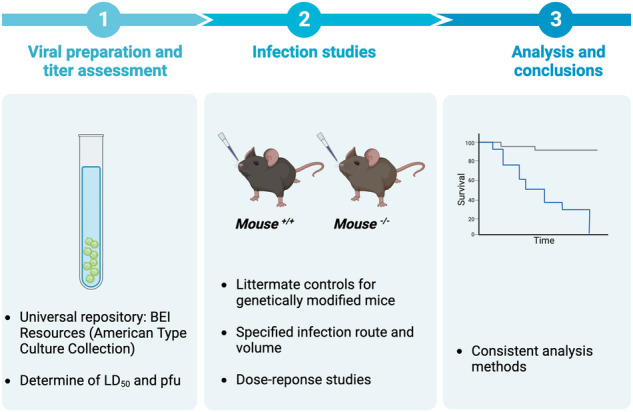
**Workflow for IAV infection studies in vivo**: Viral preparation and titer assessment should be as standardized as possible with the use of a universal repository (such as BEI resources). Infection studies in genetically modified mice and littermate controls should include dose-response experimentation, following a defined infection route and inoculation volume. Data are generated by consistent analysis methods.

**Workflow for IAV infection studies in vivo**: Viral preparation and titer assessment should be as standardized as possible with the use of a universal repository (such as BEI resources). Infection studies in genetically modified mice and littermate controls should include dose-response experimentation, following a defined infection route and inoculation volume. Data are generated by consistent analysis methods.

## Implication of pattern recognizing receptors and cell death modalities during IAV infection

Influenza A virus (IAV) infection of epithelial cells of the upper and lower respiratory tract and lung parenchyma leads to cell death contributing to inflammation and barrier loss [1]. Besides apoptosis, necroptosis and pyroptosis are triggered both in epithelial and immune cells [2, 3] leading to an inflammatory response by releasing proinflammatory cytokines and danger-associated molecular patterns (DAMPs) [4]. IAV is recognized by the innate immune system by at least three distinct classes of pattern-recognition receptors (PRRs) including the Toll-like receptors TLR3, −7 and −8 (recognizing IAV nucleic acids), the retinoic acid-inducible gene I (RIG-I) (recognizing the 5′- triphosphate RNA) and the NOD-like receptor family member NOD-, LRR- and pyrin domain-containing 3 (NLRP3) (recognizing different stimuli) [5, 6]. The activation of TLR3 by IAV dsRNA activates the production of nuclear factor-κB (NF-κB)-dependent cytokines and of type I and III interferons (IFN) [6]. Viral RNA in the cytosol is recognized by RIG-I which activates the mitochondrial antiviral signaling protein (MAVS), inducing the production of pro-inflammatory cytokines and type I and III IFN [7]. The matrix 2 (M2) proton-selective ion channel of IAV at the Golgi apparatus initiates the assembly of the NLRP3 inflammasome which leads to the caspase-1-mediated activation of interleukin-1β (IL-1β) and IL-18 [8]. Caspase-1 also cleaves and activates gasdermin D (GSDMD) resulting in pyroptosis. Z-DNA binding protein 1 (ZBP1) is another innate immune sensor that recognizes Z-RNA generated during IAV-infection leading to RHIM-mediated recruitment and activation of receptor interacting kinase 3 (RIPK3) followed by apoptosis and necroptosis [9]. During IAV-infection ZBP1 can also engage pyroptosis by caspase-1 activation and gasdermin D (GSDMD) pore formation [10] (Supplementary Figure 1).

## RIPK3 at crossroad between apoptosis and necroptosis during IAV infection

In mouse and human the RIPK1, RIPK3, ZBP1 and TIR-domain containing adaptor protein inducing interferon-β (TRIF) are the only four proteins containing a RHIM, allowing homotypic protein-protein interactions and initiating the assembly of the necrosome [11]. The necrosome contains the phosphorylated forms of RIPK3 and RIPK1, as well as FADD, caspase-8 and MLKL [12]. In IAV-infected mouse fibroblasts and alveolar epithelial cells, RIPK3 can mediate either necroptosis or apoptosis, pending on the availability of the necrosome constituting proteins [13]. The RHIM and kinase activity of RIPK3 is required to phosphorylate and activate downstream MLKL, while the RHIM, but not the kinase activity, is needed for apoptosis. In the context of apoptosis, RIPK3 recruits RIPK1 through RHIM-RHIM interactions and RIPK1 then acts as the scaffold downstream of ZBP1 to recruit FADD and caspase-8 [13]. Mouse fibroblasts and alveolar epithelial cells lacking RIPK3 cells are almost completely resistant to IAV-induced cell death compared to WT cells [13]. ZBP1 deletion in fibroblasts, respiratory epithelial cells and macrophages leads to complete cell death resistance, compared to RIPK3 deletion [13, 14, 15] suggesting that ZBP1 can also induce cell death independetly from RIPK3, most likely by directly engaging RIPK1-FADD-Caspase-8-mediated apoptosis [13].

## In vivo IAV infection studies: variables that may affect the outcome

### Role of different cell death modulators and modalities

Cell death plays a crucial role in limiting viral spread and immune activation but if uncontrolled it can contribute pathogenic inflammation and increased lung pathology [4]. In vivo*, Ripk3*^*−/−*^ mice and *Zbp1*^*−/−*^ mice were reported to be more susceptible to IAV PR8 (H1N1 strain) infection compared to wild type mice, exhibiting elevated pulmonary viral load and heightened morbidity and mortality [9, 15, 16]. Apart from inducing cell death, RIPK3 may regulate type I IFN signaling at both the transcriptional and post-transcriptional levels to inhibit viral replication and protect the host against influenza infection [17]. In contrast, *Ripk3* deficiency promotes survival of mice infected with H7N9 influenza virus [18]. ZBP1/RIPK3/RIPK1/Caspase-8-mediated apoptosis limits IAV without the need for necroptosis, but in the absence of apoptosis by a mutant *Casp8*^*DA*^ allele preventing caspase-8 autoprocessing, necroptosis may also function as an independent, “stand-alone” cell death mechanism in antiviral host defense [19]. We observed that the requirement of RIPK3 for protection against IAV PR8 (H1N1) infection in vivo is only apparent within a limited dose range of IAV challenge [17]. Moreover, this protective outcome is independent from RIPK3 kinase activity and from MLKL, demonstrating that the scaffolding function of RIPK3 rather than its kinase activity is required for protection. Altogether, this suggests that a RIPK3 function independent of necroptosis is implicated in protection against IAV-associated pathology [16] in line with the prominent role of apoptosis [19].

Apparently, the involvement of necroptosis in the protection against IAV in vivo is only observed in conditions where apoptosis is prevented by blocking caspase-8 activity or deleting of *Fadd* [13, 16, 19]. Since RIPK3 as a scaffold can be involved in apoptosis [20] while as a kinase it promotes necroptosis, it is important to also evaluate the contribution of MLKL as the final executioner of necroptosis [13, 19]. Interestingly, several studies showed that *Mlkl*^*−/−*^ mice in conditions without deletion of caspase-8 are equally susceptible to IAV compared to *Mlkl*^*+/+*^ littermates, regardless of the IAV dose of mouse adapted A/Puerto Rico/8/34 (H1N1) (PR8) strain [13, 16, 19, 21], confirming that apoptosis is the primary modality to protect against IAV infection. However, two independent studies reported that MLKL deficiency increased the protection in mice infected with a lethal dose of PR8 [22] or with A/California/7/2009 virus strain [23]. In these two studies, however, the WT controls were different: either C57BL/6J mice (The Jackson Laboratory) or C57BL/6 N (#B6NTac) mice were used as controls. Another group reported that *Mlkl*^*−/−*^ mice display similar survival and body weight loss as WT mice when challenged with PR8 virus [21]. A recent study showed that 70% of the *Mlkl*^-/-^ mice recovered after lethal infection with IAV PR8 strain (H1N1) compared to WT controls, demonstrating that necroptosis contributes to detrimental inflammation during severe infections. Indeed, therapeutic treatment of mice with a newly developed RIPK3 kinase inhibitor improved lung function, decreased inflammation and prevented mortality [22]. However, it could noted from the same paper that also p38MAPK, JAK2 and JAK3 are targeted by the RIPK3 inhibitor at 0.5 µM, which suggests a combined contribution of the targeted kinases in the improved lung function following severe IAV infection.

Moreover, we here document that the kinase activity of RIPK3 is not required for protection against IAV PR8 in vivo, confirming that necroptosis is not the main cell death mechanism implicated [16]. In line with this observation, we also showed that *Fadd*^*−/−*^ *Ripk3*^*−/−*^ mice are even more susceptible than their WT *Ripk3*^*+/+*^ littermates regardless of the IAV inoculum doses that were used, suggesting an important role for FADD-mediated apoptosis independent of RIPK3 in the control of IAV-associated mortality [16]. It should be noted that *Fadd*^*−/−*^ *Ripk3*^*−/−*^ mice develop autoimmune lymphoproliferative syndrome due to a failure of mature T cells to undergo Fas-mediated apoptosis [24]. An alternitive explanation for the extreme susceptibility of these mice may be related to a disruption in T cell function. Moreover, *Casp8*^*DA*^ *Mlkl*^*−/−*^ *Fadd*^*−/*−^ and *Casp8*^*DA*^ *Mlkl*^*−/−*^ mice were more susceptible to IAV-associated morbidity compared to WT controls, confirming that caspase-8-mediated apoptosis is crucial for protection against IAV [13, 19]. Finally, Cellular Inhibitors of ApoPtosis (cIAPs) are E3 ligases that keep RIPK1 in the survival mode of signaling [25]. Mice deficient in cIAP2 exhibit increased susceptibility and mortality to IAV infection, which can be rescued by pharmacological RIPK1 kinase inhibition or *Rip3*-deficiency [26], suggesting that sensitization to cell death death can also worsen the outcome.

Concerning the involvement of the NLRP3 inflammasome, there are also conflicting results in *Nlrp3*^*−/−*^ mice infected with IAV. It was shown that *Nlrp3* deficiency results in the susceptibility of the mice infected with PR8 [27], but *Nlrp3*^*−/−*^ mice were more likely to survive than the WT controls when infected with influenza A/Shanghai/4664 T/2013 (H7N9) strain [28].

### Virus strains

Looking in more detail at the in vivo IAV studies investigating the role of RIPK3 reveals that the different outcomes or involvement of particular cell death pathways partially could also be explained by differences in the IAV preparation, dose and route of inoculation of the mice (Table 1).

**Table 1:** Overview of the different in vivo experimental settings to assess IAV infection susceptibility in genetically modified mice. Overview of published IAV infection models using distinct genetic mouse models and infection protocols.

Publication	Major findings	Genetic mouse model and outcome	Control mice	Virus strain and generation	IAV preparation	IAV challenge dose	IAV administration route and volume
Thomas et al. [27]	IAV infection triggers the NLRP3 inflammasome to maintain respiratory integrity of the damaged lung	***Nlrp3***^***-/-***^ *mice were more susceptible than WT mice*	WT C57BL/6J mice purchased from the Jackson Laboratories	A/Puerto Rico/8/1934 (PR8, H1N1) generated by reverse genetics (Hoffmann et al., 2002)	Stocks were propagated no more than twice by allantoic inoculation of 10-day-old embryonated hen’s eggs with seed virus diluted 1:10^6.^ Virus titers expressed as 50% egg infectious dose (EID_50_)	2000-8000 EID_50_	Intranasally; 30 μL of allantoic virus stock diluted in endotoxin-free PBS
Rodrigue-Gervais IG et al. [26]	RIPK3-mediated necroptosis protects from IAV infection through maintenance of pulmonary tissue homeostasis	***Ripk3***^***-/-***^ and **WT** mice equally susceptible to a sublethal IAV dose	Source of **WT** mice not mentioned	Purified PR8 (H1N1)	Viral preparation not mentioned; viral titers determined by hemagglutination (HA) assay and expressed in hemagglutinating units (HAUs)	Sublethal 0.4xLD_50_; 12,9 HAU/mouse	Intranasally; 15 µl inoculum
Nogusa et al. [13]	RIPK3 activates in parallel necroptosis and apoptosis to destroy the infected cell, thus protecting the host	***Ripk3***^***-/-***^ mice increased susceptibility to IAV compared to ***Ripk3***^***+/+***^ controls	***Ripk3***^***+/+***^ mice	PR8 (H1N1): IAV and IBV strains	PR8 was generated by reverse genetics, propagated by allantoic inoculation of embryonated hen’s eggs with diluted (1:10^6^) seed virus. Virus titers were expressed as 50% egg infectious dose (EID_50_)	4,000 EID_50_/mouse	Intranasally (volume not mentioned)
Ren et al. [28]	The NLRP3 inflammasome is beneficial for the host to control H7N9 associated lethal pathogenesis	***Nlrp3***^*−/−*^ mice survived significantly better than the WT controls	**C57BL/6** mice purchased from the B&K Universal Group Limited (Shanghai, China)	Influenza A/Shanghai/4664 T/2013(H7N9) strain (GenBank No. KC853225-KC853232)	The viral titer was measured via tissue culture infective dose (TCID_50_) assay	5 × 10^4^ TCID_50_ with the H7N9 virus	Intranasally, 50 µl
Downey et al. [17]	RIPK3 regulates type I IFN signaling to block IAV viral replication, protecting the host	***Ripk3***^***-/*-**^ mice increased susceptibility to sub-lethal IAV compared to **WT** controls	**C57BL/6** mice (background not mentioned)	PR8 (H1N1) used for in vivo studies; H3N2 A/Hong-Kong/1/68 strain for in vitro infection of human cells	Not mentioned. Virus provided by Dr. Jonathan A. McCullers (St. Jude Children Research Hospital)	50* or 90 pfu/mouse	Intranasally; 25 µl inoculum
Oltean et al. [16]	RIPK3 platform function and necroptosis-independent is required for protection against medium IAV doses	***Ripk3***^***-/****-*^ mice increased susceptibility to medium doses of IAV compared to ***Ripk3***^***+/+***^ controls	***Ripk3***^***+/+***^ littermate mice	PR8 (H1N1) strain	PR8 was propagated in MDCK cells and viral titers were assessed by plaque assay and expressed as plaque forming units (pfu)	Very low IAV dose (0.05x LD_50_; 4 pfu); low IAV dose (0.1x LD_50_; 8 pfu); medium IAV dose (0.2x LD_50_; 16 pfu; high IAV dose (0.5x LD_50_; 40 pfu)	Intranasally; 50 µl inoculum
Gautam et al. [22]	RIPK3 inhibitor, blocked IAV-triggered necroptosis in alveolar epithelial cells which ameliorated lung inflammation and prevented mortality after lethal IAV dose	**WT** mice treated with the RIPK3 kinase inhibitor UH15-38 are protected from lethal IAV infection.	WT C57BL/6 mice (from Taconic)	PR8 (H1N1) and A/California/04/2009 (H1N1)	Mouse-adapted Influenza A/Puerto Rico/8/1934 and and A/California/04/2009 were propagated by allantoic cavity inoculation of 10-day embryonated chicken egg. Virus titers were determined by plaque assay on MDCK cells obtained from ATCC.	Lethal dose of PR8 (6,000× EID_50_)	Intranasally; volume not mentioned
Thapa et al. [15]	DAI senses IAV and binds to RIPK 3 triggering apoptosis and necroptosis of infected cell, thus protecting the host	***Zbp1***^***-/-***^ mice were more susceptible to IAV compared to ***Zbp1***^***+/+***^ mice	***Zbp1***^***+/+***^ mice	IAV strains used: PR8 (H1N1), Brisbane/59/2007(H1N1), Brisbane/10/ 2007(H3N2), and Perth/16/2009 (H3N2) and two strains of influenza type B: B/Brisbane/60/2008 and B/Florida/4/2006 PR8 (H1N1) used for in vivo experiments IAV strain PR8 and A/HKx31were generated by reverse genetics (Hoffmann et al., 2002).	PR8 was generated by reverse genetics, propagated by allantoic inoculation of embryonated hen’s eggs with diluted (1:10^6^) seed virus. Virus titers were determined as 50% egg infectious dose (EID_50_)	1000 EID_50/_ mouse	Intranasally (volume not mentioned)
Kuriakose et al. [14]	ZBP1 senses IAV triggering cell death and inflammatory responses via the RIPK1–RIPK3–Caspase-8 axis	***Zbp1***^***-/-***^ mice were more protected against IAV infection compared to **WT** mice	Source of **WT** mice not mentioned	Influenza A/X31 (H3N2), PR8, and the non- mouse adapted seasonal strains influenza A/Brisbane/59/2007 (H1N1) and A/Switzerland/ 9715293/2013 (H3N2) PR8 (H1N1) was used for in vivo experiments PR8 (H1N1) was generated by an eight-plasmid reverse genetics system	PR8 was generated by reverse genetics, propagated by allantoic inoculation of embryonated hen’s eggs with diluted (1:10^6^) seed virus. Virus titers were expressed as 50% egg infectious dose (EID_50_)	1000 pfu (aprox. 1x LD_50_)	Intranasally 30 µl inoculum
Momota et al. [32]	IAV-induced ZBP1 mediates inflammasome-independent production of IL-1β and neutrophil extracellular traps (NETs) resulting in either protective or pathological outcomes in vivo	***Zbp1***^***-/-***^ mice were more susceptible to IAV compared to ***Zbp1***^***+/-***^ mice when challenged intranasally, but they showed less mortality when challenged intratracheally	***Zbp1***^***+/-***^ mice	Virus preparation PR8 strain (Koyama et al.,2010).	PR8 was propagated in MDCK cells and viral titers were assessed by plaque assay	50 pfu; 1x LD50 in 30 μl of PBS i.n. 50 pfu; 1x LD50 in 50 μl of PBS i.t.	Intranasally; 30 μl inoculum or intratracheally; 50 μl inoculum
Nogusa et al. [13]		***Mlkl***^***-/-***^ mice were equally susceptible to IAV challenge as ***Mlkl***^***+/+***^ mice (aprox. 75% survival)	***Mlkl***^***+/+***^ mice	PR8 (H1N1): IAV and IBV strains	PR8 was generated by reverse genetics, propagated by allantoic inoculation of embryonated hen’s eggs with diluted (1:10^6^) seed virus. Virus titers were expressed as 50% egg infectious dose (EID_50_)	4,000 EID_50_/mouse	Intranasally (volume not mentioned)
Nogusa et al. [13]		***Mlkl***^***-/-***^***Fadd***^***-/-***^ mice were more susceptible as ***Mlkl***^***-/-***^ mice	***Mlkl***^***+/+***^ and ***Mlkl***^***-/-***^ mice	PR8 (H1N1): IAV and IBV strains	PR8 was generated by reverse genetics, propagated by allantoic inoculation of embryonated hen’s eggs with diluted (1:10^6^) seed virus. Virus titers were expressed as 50% egg infectious dose (EID_50_)	4,000 EID_50_/mouse	Intranasally (volume not mentioned)
Gautam et al. [22]		***Mlkl***^***-/****-*^ mice increased survival to lethal dose of IAV compared to **WT** controls	WT C57BL/6 mice (from Taconic)	PR8 (H1N1) strain	Mouse-adapted strains of Influenza A/Puerto Rico/8/1934 were propagated by allantoic cavity inoculation of 10-day embryonated chicken egg. Virus titers were determined by plaque assay on MDCK cells obtained from ATCC.	Lethal dose of PR8 (6,000× EID_50_)	Intranasally; not mentioned
Shubina et al. [19]	IAV activates ZBP1- RIPK3-mediated necroptosis and apoptosis in infected cells, promoting host defense	***Casp8***^***DA***^ mice are equally susceptible to IAV infection as **WT** mice	**WT littermate** controls or **WT C57BL/6** mice (The Jackson Laboratory)	PR8 (H1N1) strain	PR8 was generated by reverse genetics, propagated by allantoic inoculation of embryonated hen’s eggs with diluted (1:10^6^) seed virus. Virus titers were expressed as 50% egg infectious dose (EID_50_)	2500 EID_50_/ mouse	Intranasally (volume not mentioned)
Shubina et al. [19]		***Casp8***^***DA***^ ***Mlkl***^***-/-***^ mice are as susceptible to IAV infection as **WT** mice	**WT littermate** controls or **WT C57BL/6** mice (The Jackson Laboratory)	PR8 (H1N1) strain	PR8 was generated by reverse genetics, propagated by allantoic inoculation of embryonated hen’s eggs with diluted (1:10^6^) seed virus. Virus titers were expressed as 50% egg infectious dose (EID_50_)	2500 EID_50_/ mouse	Intranasally (volume not mentioned)
Zhang et al. [9]	IAV activates ZBP1 initiating RIPK3-MLKL -dependent necroptosis. This leads to inflammatory pathology upon IAV infection	***Zbp1***^***−/−***^ displayed increased mortality compared to WT controls	**WT** controls **C57BL/6J mice** (The Jackson Laboratory)	PR8 (H1N1) strain	IAV strains were propagated by allantoic inoculation of embryonated hen’s eggs with diluted (1:10^6^) seed virus. Virus titers were determined as 50% egg infectious dose (EID_50_) and by plaque assay on Madin-Darby Canine Kidney (MDCK) cells	Modestly lethal IAV dose (2,500 EID_50_ ~LD20) and lethal dose 6000 EID_50_)	Intranasally (volume not mentioned)
Zhang et al. [9]		***Ripk3***^*−/−*^ showed increased mortality compared to WT controls	**WT** controls **C57BL/6J mice** (The Jackson Laboratory)	PR8 (H1N1) strain	IAV strains were propagated by allantoic inoculation of embryonated hen’s eggs with diluted (1:10^6^) seed virus. Virus titers were determined as 50% egg infectious dose (EID_50_) and by plaque assay on Madin-Darby Canine Kidney (MDCK) cells	Modestly lethal IAV dose (2,500 EID_50_ ~LD_20_) and lethal dose 6000 EID_50_)	Intranasally (volume not mentioned)
Zhang et al. [9]		***Mlkl***^***-/-***^ displayed same mortality to WT controls for the modestly lethal dose and increased survival compared to WT for the lethal IAV dose	**WT** controls **C57BL/6J mice** (The Jackson Laboratory)		IAV strains were propagated by allantoic inoculation of embryonated hen’s eggs with diluted (1:10^6^) seed virus. Virus titers were determined as 50% egg infectious dose (EID_50_) and by plaque assay on Madin-Darby Canine Kidney (MDCK) cells	Modestly lethal IAV dose (2,500 EID_50_ ~LD_20_) and lethal dose 6000 EID_50_)	Intranasally (volume not mentioned)
Oltean et al. [16]		***Mlkl***^***-/-***^ mice were equally susceptible to different IAV doses as ***Mlkl***^***+/+***^ controls	***Mlkl***^***+/+***^ mice	PR8 (H1N1) strain	PR8 was propagated in MDCK cells and viral titers were assessed by plaque assay and expressed as plaque forming units (pfu)	Very low IAV dose (0.05x LD_50_; 4 pfu); low IAV dose (0.1x LD_50_; 8 pfu); medium IAV dose (0.2x LD_50_; 16 pfu; high IAV dose (0.5x LD_50_; 40 pfu)	Intranasally; 50 µl inoculum
Oltean et al. [16]		***Ripk3***^***-/-***^ ***Fadd***^***-/-***^ mice were far more susceptible to different IAV doses compared to ***Ripk3***^***+/+***^ ***Fadd***^***+/+***^ *and to****Ripk3***^***-/-***^ controls	***Ripk3***^***+/+***^ ***Fadd***^***+/+***^ *and****Ripk3***^***-/-***^ *mice*	PR8 (H1N1) strain	PR8 was propagated in MDCK cells and viral titers were assessed by plaque assay and measured as plaque forming units (pfu)	very low IAV dose (0.05x LD_50_; 4 pfu); low IAV dose (0.1x LD_50_; 8 pfu); medium IAV dose (0.2x LD_50_; 16 pfu; high IAV dose (0.5x LD_50_; 40 pfu)	Intranasally; 50 µl inoculum
Yi-Han et al. [23]	MLKL deficiency improves antioxidant and mitochondrial activity upon IAV infection	***Mlkl***^***-/-***^ mice showed increased survival and reduced weight loss compared to WT mice	*C57BL/6N (#B6NTac) mice*	Pandemic Influenza A/California/7/2009 and PR8 (H1N1) strain	Not mentioned	250 pfu	Intranasally; volume not mentioned
Lei et al. [21]	MLKL is dispensable for host inflammatory responses to IAV in vivo	***Mlkl***^***-/-***^ mice showed similar survival and body weight loss as WT mice	WT C57BL/6J and C57BL/6N (Jackson Laboratory)	PR8 (H1N1) strain	PR8 was grown in SPF embryonated chicken eggs and was purchased from Charles River Laboratories.	25 pfu	Intranasally, 25 µl inoculum
Gautam et al. [22]		***Zbp1***^***-/-***^***, Ripk3***^***-/-***^***and Ripk1***^***kinase-dead***^ mice similar susceptibility to lethal dose of IAV compared to **WT** controls.	WT C57BL/6 mice (from Taconic)	PR8 (H1N1) strain	Mouse-adapted strains of Influenza A/Puerto Rico/8/1934 were propagated by allantoic cavity inoculation of 10-day embryonated chicken egg. Virus titers were determined by plaque assay on MDCK cells obtained from ATCC.	lethal dose of PR8 (6,000× EID_50_)	Intranasally; not mentioned

The table provides a systematic overview of the major findings in the different publications, the outcome of the transgenic mice, the control mice used in the experimental setting, the IAV preparation if mentioned or not, the IAV challenge dose, and the different outcomes on the role of cell death pathways following IAV infection and route of inoculation.

*50 pfu/mouse killed 30 % of the WT mice.

The most commonly used influenza A virus strain used to examine disease pathogenesis and the immune responses in vivo is the mouse adapted PR8 virus strain. This strain was adapted to mice by multiple serial sequential passages of A/Puerto Rico/8/34 in mouse lung. During this process, the strain acquired mutations allowing a high replication efficiency and evasion of the immune response, therefore becoming highly virulent for the mice [29]. For instance, contrary to other strains, such as H7N9, PR8 (H1N1) cannot infect macropahges [29].

Viral preparations are mostly generated by allantoic inoculation of embryonated eggs or by propagation in Madin-Darby canine kidney (MDCK) cells. Embryonated chicken eggs are the standard procedure for producing viral stocks, and has the advantage of producing high virus titers in a short period of time. The mammalian cell culture-based production of viral stocks for influenza vaccine use, such as using MDCKs, produces about 4-fold lower titers than the egg-based process, increasing manufacturing costs [30, 31]. Moreover, cell culture-based viruses have increased batch-to-batch variation and risk for mycoplasma contamination [31].

Viral titers are assessed by egg infectious dose (determine EID_50_) or by plaque froming unit assay (determine the pfu) [9, 14, 16, 19, 32]. However, some labs do not inform on the precise method of the viral preparation [17, 26]. Table 1 provides a systematic overview of the experimental settings and the different outcomes following IAV infection (Table 1). In the next paragraphs, we highlight how differences in the IAV inoculum dose, virus preparation and titration as well as the route of inoculation may impact the comparison and the conclusions.

### Viral dose of infection

**Fig. 1:**
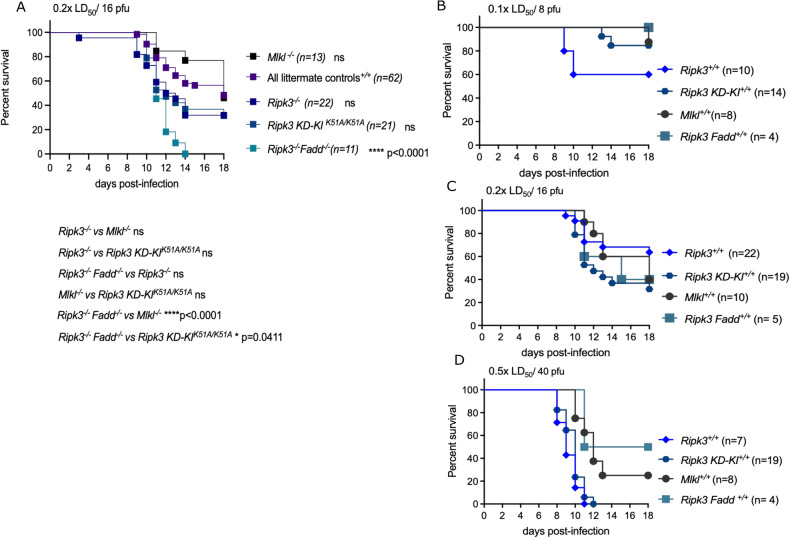
Survival of genetically modified mice compared to different littermate controls (pooled) at medium IAV challenge dose and comparison of survival different littermates challenged at different IAV doses. Pooled littermate controls (*Ripk3*^*+/+*^*, Ripk3 KD-KI*^*+/+*^*, Mlkl*^*+/+*^, and *Ripk3*^*+/+*^*Fadd* ^*+/+*^ mice) compared with the *Ripk3*^*−/−*^*, Ripk3 KD-KI*^*K51A/K51A*^*, Mlkl*^*−/−*^, and *Ripk3*^*−/−*^*Fadd* ^*−/−*^. **A** Mice were challenged with different IAV doses (here 0.2x LD_50_/16 pfu). Survival curves were plotted for indicated groups and evaluated statistically according to Kaplan–Meier (GraphPad Prism 8). ns, not significant; ****p < 0.0001 [16]. Survival of littermate controls (**B**−**D**): *Ripk3*^*+/+*^, *Ripk3 KD*-*KI*^*+/+*^, *Mlkl*^*+/+*^, and *Ripk3*^*+/+*^*Fadd* ^*+/+*^ mice at different IAV challenge doses Mice were challenged with different IAV doses (0.1 × LD_50_*/*8 pfu; 0.2 × LD_50_/16 pfu; 0.5 × LD_50_/40 pfu). Survival curves were plotted for indicated groups [16]. Mice: *Ripk3*^*−/−*^ were kindly provided by Dr. Vishva Dixit (Genentech, San Francisco) [44], *Mlkl*^*−/−*^ by Dr. Alexander Warren and Dr. James Murphy [45] and Ripk3 KD-KI^K51A/K51A^ mice by Dr. John Bertin by GlaxoSmithKline [20]. The *Ripk3*^*−/−*^ animals were congenic to the C57BL/6 N background, while all other strains were of the C57BL/6 J background, and were therefore compared with the appropriate littermate controls. *Ripk3*^*−/−*^ mice were housed in individually ventilated cages in a conventional animal house. The other mice were bred and housed in the SPF facility in individually ventilated cages. Three weeks prior to the experiment all mice were transferred to the conventional animal house and allowed to go through a quarantine and accommodation period of minimum 3 weeks before the infection experiment. Littermate controls of *Ripk3*^*−/−*^*, Mlkl*^*−/−*,^ *Ripk3*^*−/−*^*Fadd*^*−/−*^ and *Ripk3 KD-KI*^*K51A/K51A*^ were used in each experiment. In all experiments, 10–15- week-old mice were used. All animal experiments were done under conditions specified by law (European Directive and Belgian Royal Decree of November 14, 1993) and approved by the Institutional Ethics Committee on Experimental Animals. Viral infection: Age-matched mice were anesthetized with a cocktail of 87,5 mg/kg ketamine and 12,5 mg/kg xylazine intraperitoneally and infected intranasally with 50 μl/20 g phosphate-buffered saline (PBS) containing different doses of influenza virus A/PR/8/3443, as described in the legends. The plaque-forming units (pfu) were determined by plaque assay on Madin-Darby Canine Kidney (MDCK) cells [31]. The LD_50_ of the viral batch was determined on BALB/c mice and 1x LD_50_ represented 80 pfu, as determined in the lab of Prof. Saelens. Although the LD_50_ is not referring to 50% of death in the mice that were used in this study, the nomenclature is used together with the pfu to have a supplementary information regarding the power of the virus in vivo. This terminology is often used in the papers cited here. Age- and sex-matched mice were challenged with 0.05x LD_50_ (4 pfu), 0.1x LD_50_ (8 pfu), 0.2x LD_50_ (16 pfu) or 0.5x LD_50_ (40 pfu) and monitored for survival and weight loss over a period of at least 18 days. We used the following 4 scores of clinical symptoms: 0 = no visible signs of disease; 1 = slight ruffling of fur; 2 = ruffled fur, reduced mobility; 3 = ruffled fur, reduced mobility, rapid breathing; 4 = ruffled fur, minimal mobility, huddled appearance, rapid and/or labored breathing indicative of pneumonia and body temperature below 32 °C. For the combination of body weight loss by 30% and a clinical score 4 the mice were considered moribund and euthanized by CO_2_ asphyxiation or cervical dislocation (EC2016–17). The lethality includes an ethical endpoint during which euthanasia was performed. This ethical endpoint is a combination of 30% weight loss and a clinical score of 4. Survival curves: All the survival data were plotted using the Kaplan–Meier survival analysis with the software Prism 10.1.0.

Most studies use the mouse-adapted H1N1 laboratory strain of influenza A, PR8 [33]. Despite the use of the same strain, the lethal outcome following IAV infection looks often very different. One study found that the IAV infection with a dose that is expected to be sublethal for most of the control mice (0.4x LD_50_; 12.9 hemagglutination units (HAU)/ mouse) did not reveal the presumed sensitized lethality in RIPK3-deficient mice compared to their controls [26]. In contrast, other studies found that RIPK3-deficient mice were more susceptible to IAV when challenged with a dose that was sublethal for the majority of the control mice [13]. Yet, another study reported by comparing two doses that a low IAV dose (50 pfu/mouse) displayed higher lethality, while this difference is not observable with a higher challenge dose (90 pfu/mouse), likely because of the lethal outcome for the majority of the control mice [17]. This finding is in accordance with our results (see below), i.e., that RIPK3 is required for protection against IAV infection in vivo in response to a limited range of challenging dose, while at high dose it is dispensable for protection. We demonstrate that *Ripk3*^*−/−*^ mice were more susceptible than their *Ripk3*^*+/+*^ counterparts when infected with medium IAV doses (in this study corresponding to 0.2x LD_50_/16 pfu). However *Ripk3*^*−/−*^ mice were not more susceptible compared to their littermate controls when infected with very low or low doses (0.05x LD_50_/4 pfu and 0.1x LD_50_/8 pfu) and high doses (0.5x LD_50_/40 pfu) of PR8 (see Fig. 1) [16]. These results underline that the *Ripk3* phenotype is restricted to a range of infectious doses. Also, the involvement of ZBP1 in modulating lethality following IAV-infection shows various outcomes. When challenged with 1x LD_50_ of PR8*, Zbp1*^*−/−*^ mice were shown to be either better protected from IAV infection compared to their WT controls [14, 15] or to be more susceptible compared to their *Zbp1*^*+/+*^ controls [15]. Another study revealed that the infection route could explain these results. Intranasal challenge revealed that *Zbp1*^*−/−*^ mice were more susceptible to IAV compared to *Zbp1*^*+/−*^ mice, while intratracheal virus inoculum administration resulted in reduced mortality with the same IAV dose (1x LD_50_; 50 pfu) [32]. Both *Zbp1*^*−/−*^ and *Ripk3*^*−/−*^ mice displayed increased mortality compared to C57BL/6 J controls when challenged with a sublethal as well as a lethal IAV dose [9]. All these experiments underline the importance of testing a range of viral doses to address the susceptibility of genetically modified mice. It also shows that at higher infectious doses both ZBP1 and RIPK3 may contribute to immunopathology resulting in increased lethality.

Besides the range of viral dose for challenge, it is important to note that the humane endpoints for each study can vary. The maximum weight loss that are used to determine the humane endpoint beyond which the mice are euthanized are very important in determining survival numbers and impact survival curves [34]. Some institutional protocols impose euthanasia of mice that have lost 25% of their body weight relative to the time of virus inoculation. In this particular IAV infection model, mice can lose more than 30% of their weight and still recover [34]. We noticed that these humane endpoints vary between labs: some apply ethical endpoint of 30% body weight loss [9], some more than 35% [19, 32], a combination of weight loss and other clinical symptoms [16], while for other studies we were not able to retrieve this information from the methodology [4, 17, 21, 23, 26, 28].

Interestingly, *Mlkl*-deficient mice were shown to be equally susceptible to IAV as their WT littermates [13], demonstrating that the necroptotic execution mechanism is not implicated in the protective effect. However, another independent study also showed that *Mlkl*^*−/−*^ mice were equally susceptible to lethality as WT control in response to a moderate IAV dose (described as EID_2500_, approximately 1x LD_20_) but exhibited a better survival when challenged with a higher dose (EID_6000_), indicating that the presence of MLKL is associated with a worse outcome at high IAV challenge dose. In this context, MLKL was shown to drive a pathogenic recruitment of neutrophils and lethality during severe IAV disease [9]. We found, however, that different doses (0.1x LD_50_/8 pfu, 0.2x LD_50_/16 pfu, and 0.5x LD_50_/40 pfu,) of IAV did not reveal any role for MLKL neither protective nor worsening [16]. We showed that the RIPK3 kinase activity is not required for partial protection against IAV infection at different viral doses and neither is MLKL, suggesting that necroptosis is not involved in the protection against small, medium or high IAV doses in vivo [16]. However, the conclusions from these experiments might be difficult to draw, since it has been reported that RIPK3Ripk3 K51A kinase dead knock-in (Ripk3 KD-KI^K51A/K51A^) mice used here express very low levels of RIPK3 protein compared to wild type littermate [20] which may affect the scaffolding function of RIPK3. Therefore, differences in viral challenge due to non-standardized doses in titer and volume of application may explain the various outcome ranging from no [26], partial [16] to complete protection of *Ripk3*^*−/−*^ mice [13, 17].

### Viral preparation, titer assessment and route of infection

It is important that experiments performed in different laboratories can be compared in order to contribute to a better understanding of cell death induced upon IAV infection. Such a comparison relies on an exact description of the viral dose used in infection experiments. While performing these comparisons, we found that the description of the IAV challenge inoculum differs between labs (Table 1). For example, Downey and colleagues inoculated the mice with a low IAV dose defined as 50 pfu per mouse [17]. One group challenged the mice with a sublethal dose described as 0.4x LD_50_ corresponding to 12.9 hemagglutination units (HAU) [26]. Another group reported their viral titers as egg infectious dose 50 (EID_50_) [9, 13, 15]. We used the LD_50_ terminology as determined in BALB/c mice as well as the pfu determined by plaque assay [16].

Indeed, such different ways of determining the IAV doses may affect the conclusions. The infectious dose of a certain IAV preparation may be assessed by determining the 50% egg infectious dose (EID_50_), the plaque forming unit assay (pfu/mL) or the median tissue culture infectious dose (TCID_50_). We propose that viral dose for in vivo use should be standardized by cellular assays (pfu/mL) and the median lethal dose for 50% of wild type mice (LD_50_). It is also important to note that the virulence of PR8 IAV strains may vary between laboratories and the mouse strains used. A uniform source of IAV challenge strains, e.g., use of strains that can be procured from a repository such as BEI Resources, managed by the American Type Culture Collection, could also help in this process. Finally, other variables such as the volume used (30 µl, 50 µl) and the route of infection (intranasal, versus intratracheal) may affect the outcome of the experiment [32] and also requires standardization. Regarding the inoculum volume, a study has shown that mice challenged with a 25 µl inoculum volume rapidly recovered infection with a dose considered lethal when inoculated in 35 or 50 µl volumes [35]. This could be due to poor distribution and subsequent infection of the lung when using relatively small inoculum volumes (such as 20 µL) when compared to higher inoculum volumes [36].

A similar discussion regarding the roles of RIPK3, MLKL, and ZBP1 has been raised previously [37]. In this review, the authors indicated the possible outcomes of upper and lower respiratory tract administration of IAV. Human IAV strains primarily infect human epithelial cells of the upper respiratory tract and typically are associated with mild to moderate disease. Lower respiratory tract infections are associated with increased higher morbidity and mortality [37]. This is an important aspect that needs to be considered when analyzing tract-specific pathophysiology of IAV, respiratory epithelial damage, and inflammation.

## Littermate controls and genetic backgrounds

From Table 1 it is obvious that an evenness in the use of littermate controls and comparison between appropriate genetic backgrounds in the experimental setup is also essential for reliable conclusions between genetically modified mouse strains [9, 38]. Littermates have comparable genetic background to the knock-out (KO) mice and are exposed to the same environment during development and birth. C57BL/6 N ES cells have been an important source for the construction of transgenic mice by the International Mouse Phenotyping Consortium (IMPC) [39] while some spontaneous mutations on the C57BL/6 J background are used in many biomedical studies [40]. It is also important to specify the genetic background of the transgenic mice since both C57BL/6 N and C57BL/6 J ES cells have been used [41]. If applicable, also the extend of backcrossing in the C57BL/6 N and C57BL/6 J strains should be mentioned or even quantified [42]. Indeed, genetic differences between C57BL/6 J (B6J) and C57BL/6 N (B6N) sub-strains affect the susceptibility to and pathology associated with IAV infection, with B6J mice being significantly more susceptible to A/California/04/09 H1N1 virus (H1N1), A/Vietnam/1203/2004 (H5N1), and A/Anhui/1/2013 (H7N9) virus strains compared to 6NJ [43].

To address the issue of different genetic background of various transgenic mice we wondered whether pooling the different littermate controls from our previous study [16] and comparing them with the different genetically modified mice challenged with the same IAV dose, would affect the interpretation of the experimental outcomes. To illustrate the effect of genetic backgrounds, we merged the IAV infection results from all control mice (*Ripk3*^*+/+*^*, Ripk3 KD-KI*^*+/+*^*, Mlkl*^*+/+*^, and *Ripk3*^*+/+*^*Fadd* ^*+/+*^) and compared these for each IAV challenge dose (0.1x LD_50_/8 pfu/mouse; 0.2x LD_50_/16 pfu/mouse; 0.5x LD_50_/40 pfu/mouse) with the separate groups of *Ripk3*^*−/−*^*, Ripk3 KD-KI*^*K51A/K51A*^*, Mlkl*^*−/−*^, and *Ripk3*^*−/−*^*Fadd* ^*−/−*^ transgenic mice (Fig. 1 A). By doing so, we found that, compared to the pooled control mice, *Ripk3*^*−/−*^ mice showed a non-significant difference for the medium dose, contrarily to the control mice as shown previously (Fig. 1A). This is probably observed because the *Ripk3 KD-KI*^*+/+*^ *and Mlkl*^*+/+*^ mice apparently are more resistant to the same IAV dose compared to the *Ripk3*^*+/+*^ mice [16]. The variance in survival between the different wild type littermates for all IAV challenge doses are shown in Fig. 1B−D [16]. The results of Fig. 1 emphasize again the need to include proper control mice to compare the experimental outcome of a genetic modification.

## Conclusions

The complexity of the multiple cell autonomous (cell survival, apoptosis, necroptosis, pyroptosis) and intercellular responses by immune cells, and the amplification of a virus should be carefully evaluated in vivo when comparing the outcome of loss-of-function mutations through by comparing the outcome of IAV challenge in genetically modified with control mice. First, a defined set of different viral doses should be tested in genetically modified mice to investigate their susceptibility. Second, standardized viral preparation, titer assessment, dose-response challenge experiments and a standardized route of administration are crucial to enable cross-comparisons of reported data in order to draw solid conclusions. Finally, the use of littermate controls and matched housing background for the mice are the appropriate controls in these settings to avoid inaccurate conclusions.

### Supplementary information

Supplementary figure 1

Supplementary figure 1 legend
