# Supplementary figures and images for: Need for standardization of Influenza A virus-induced cell death in vivo to improve consistency of inter-laboratory research findings
Source: Cell Death Discov. 2024 May 22;10:247. doi: 10.1038/s41420-024-01981-w (PMC11111761; doi:10.1038/s41420-024-01981-w)

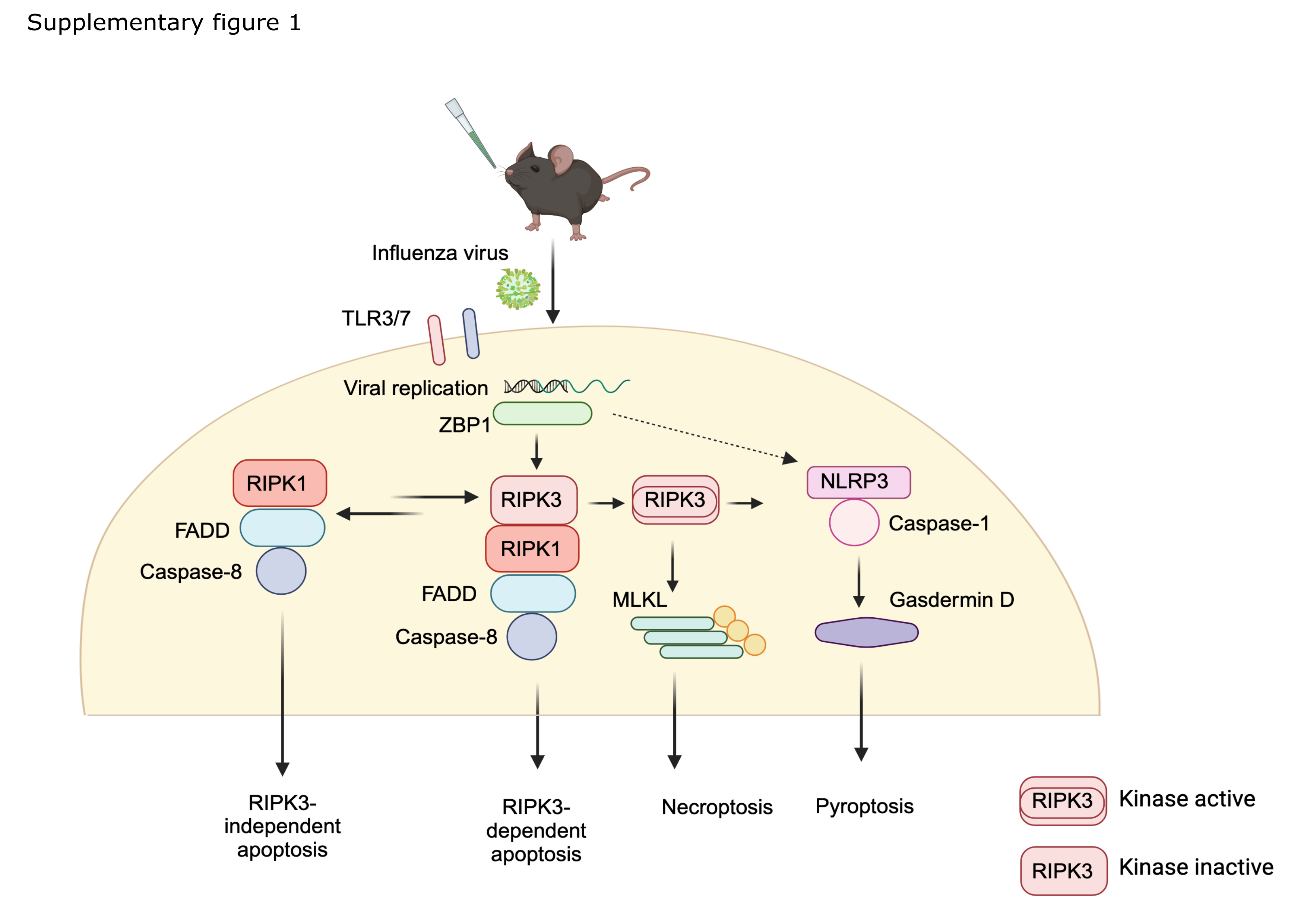

Supplement: Supplementary file 1 — Supplementary figure 1 [file 41420_2024_1981_MOESM1_ESM.tif]
