# Supplementary material for: Need for standardization of Influenza A virus-induced cell death in vivo to improve consistency of inter-laboratory research findings
Source: Cell Death Discov. 2024 May 22;10:247. doi: 10.1038/s41420-024-01981-w (PMC11111761; doi:10.1038/s41420-024-01981-w)
Supplement: Supplementary file 2 — Supplementary figure 1 legend [file 41420_2024_1981_MOESM2_ESM.docx]

**Supplementary Figure 1. Proposed cell death mechanisms induced by influenza A virus.** Once IAV enters the cell it starts replicating. IAV-derived Z-RNA is sensed by ZBP1 which can directly bind to RIPK3 (47)(15). Downstream of ZBP1, RIPK3 initiates two parallel pathways of cell death: either by associating with RIPK1, FADD and caspase-8 driving apoptosis (RIPK3 kinase inactive) or by associating and phosphorylating its downstream target, MLKL leading to necroptosis (RIPK3 kinase active)(13)(17)(23). In macrophages, ZBP1 can also engage pyroptosis by NLRP3 and caspase-1 activation and the gasdermin D pore formation. More research needs to determine the exact interaction between ZBP1 and NLRP3 (10).
